# High-Biofidelity Biomodel Generated from Three-Dimensional Imaging (Cone-Beam Computed Tomography): A Methodological Proposal

**DOI:** 10.1155/2020/4292501

**Published:** 2020-01-28

**Authors:** Rosa Alicia Hernández-Vázquez, Guillermo Urriolagoitia-Sosa, Rodrigo Arturo Marquet-Rivera, Beatriz Romero-Ángeles, Octavio Alejandro Mastache-Miranda, Juan Alejandro Vázquez-Feijoo, Guillermo Urriolagoitia-Calderón

**Affiliations:** ^1^Universidad Politécnica del Valle de México, Av. Mexiquense s/n esquina Av. Universidad Politécnica, Col. Villa Esmeralda, Tultitlán 54910, Estado de México, Mexico; ^2^Instituto Politécnico Nacional, Escuela Superior de Ingeniería Mecánica y Eléctrica, Sección de Estudios de Posgrado e Investigación Unidad Profesional Adolfo López Mateos, Zacatenco Edificio 5, 2 piso Col. Lindavista, Delegación Gustavo A. Madero, Mexico City 07320, Mexico

## Abstract

Experimental research on living beings faces several obstacles, which are more than ethical and moral issues. One of the proposed solutions to these situations is the computational modelling of anatomical structures. The present study shows a methodology for obtaining high-biofidelity biomodels, where a novel imagenological technique is used, which applies several CAM/CAD computer programs that allow a better precision for obtaining a biomodel, with highly accurate morphological specifications of the molar and tissues that shape the biomodel. The biomodel developed is the first lower molar subjected to a basic chewing simulation through the application of the finite element method, resulting in a viable model, able to be subjected to various simulations to analyse molar biomechanical characteristics, as well as pathological conditions to evaluate restorative materials and develop treatment plans. When research is focused in medical and dental investigation aspects, numerical analyses could allow the implementation of several tools commonly used by mechanical engineers to provide new answers to old problems in these areas. With this methodology, it is possible to perform high-fidelity models no matter the size of the anatomical structure, nor the complexity of its structure and internal tissues. So, it can be used in any area of medicine.

## 1. Introduction

Traditionally, it is considered that the main tool of medicine has been biology. Biological sciences have been able to explain the behaviour of cells, tissues, and organs [1]. Nevertheless, research in the areas of health may face large and varied constraints that make it difficult to carry out. Experiments directly including living beings involve ethical and economic issues [2] and situations that may be risky [3, 4] for both the individuals that are been studied and the research staff [5]. Given these circumstances, it is increasingly common to find the implementation of numerical models that can be approximated to real situations and necessities [6, 7]. The results and solutions depend on the quality of biomodels, implementation of experimental data, and clinical application [8].

Modelling simulation of biological systems by the finite element method currently occupies an important new area to be used in various areas of dentistry [9–11] from operative dentistry to implantology [12]. This has made it possible to change paradigms and misconceptions on the mechanical properties of dental tissues [13, 14]. This allows us taking better clinical decisions in prevention, diagnosis [15, 16], and treatment plans [17], as well as finding new materials [18, 19] and fixtures [20–22] on the conditions of the stomatognathic system [23, 24] on major areas of action and to study odontology. And this is an entity of great structural complexity, dynamism, and constant change.

The design of models of the human body is basically divided into three levels: geometric, physical, and physiological [25]. The geometric level aims to accurately describe the anatomy of the tissues or organs to be analysed in terms of its shape, dimensions, and structures that constitute them. This requires the acquisition of images of the areas of interest and is accomplished through the use of clinical imaging (CT, MRI, ultrasound, etc.).

The data are obtained in a DICOM format (Digital Imaging and Communication in Medicine); then, these data are exported to computer programs of CAM/CAD type (Computer-Aided Design/Computer-Aided Manufacturing). The physical modelling intends to reproduce mechanobiological and/or biomechanical tissues and organs. Once the geometric patterns are obtained, they are subjected to analysis by programs using mainly the finite element method or boundary method. Finally, the physiological level seeks the reproduction of the functional phenomena of tissues and organs, as well as their analysis within the system to which they belong. It is also possible to use them to analyse pathologies and other phenomena that modify the physiology [26].

It is worth mentioning that there are some aspects that must be considered to achieve the modelling of anatomical structures, such as dental organs. One of the main aspects is the amount of time required for designing and obtaining the necessary volumetric image, which is then subjected to analysis. This step is very important because when the anatomical structure is reproduced more faithfully, greater accuracy is possible for analysis [27].

Actually, there are several programs of computer-aided simulation (CAM) that enable imaging of various anatomical structures three-dimensionally. These programs are able to provide excellent reproducibility of details. However, a fine reproduction of dental morphological features (the crown and roots) is limited by the method of data acquisition and memory capacity demanded by modelling [28]. Therefore, the difficulty to model these structures depends on three main factors.

The quality of the DICOM images depends on the ability of the imaging equipment, the training necessary to manage these programs, and morphology knowledge of the specific area that is seeking the model. These factors allow obtaining a very detailed and accurate three-dimensional model of the anatomical shape for both the outline and the internal geometry. That is why, it is so important to have a methodology that facilitates access to develop tools for a more accurate biomodelling. The present work shows a methodology to obtain a biomodel for first lower molar, considering all the above factors.

## 2. Methodology

To acquire images of the molar, a digital volume tomography (DVT) of the maxilla and mandible with the system of cone-beam computed tomography (CBCT), through which DICOM images were obtained, is performed. This system is used for image visualization of hard tissue. It is widely used in medicine and dentistry in the cranial-facial region. The CBCT provides high-quality level resolution submillimetre images with excellent diagnostic visualization [29]. Tomographic slices are at time intervals from 10 to 70 seconds, which makes the radiation dose fifteen times lower than the conventional computed tomography [30]. In this way, the evaluation capacity in the dental clinic is increased, with less distortion of images from 3D [31].

This new modality of imaging test provides accurate and high-quality images of the bone elements in the maxillofacial complex three-dimensional representation. Unlike conventional tomography that shows consecutive cuts, the data obtained by a TVD and processed by a computer are used in the reconstruction of the studied volume. TVD is composed of voxels (which are three-dimensional pixels), which allows for dynamic analysis of information to simultaneously scan the object in cross, frontal, or sagittal direction.

In this manner, the structures that are not visible on a standard two-dimensional X-ray are disclosed. In the tomographic study, Batex model Ez3D digital volume tomography is used, which has a kVp of 90.0 mA (3.8 beam intensity, and 477 images or cuts with a distance (slide thickness of 0.5 mm between them are obtained. The spaces between pixels (pixel spacing) are at a ratio of 0.3:0.3 mm.

**Figure 1:**
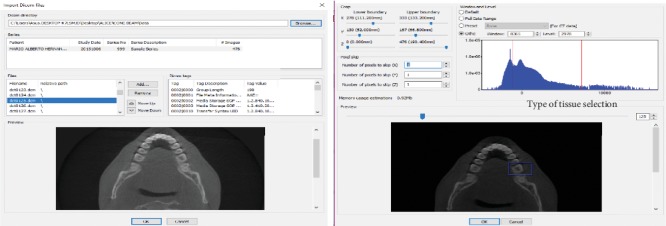
Delimitation of the area of interest.

The DICOM images obtained from the tomography were imported to the ScanIP® computer program for segmenting images to obtain a 3D virtual biomodel. Segmentation is the process of separating structures that must be represented in the biomodel. Once the DICOM files are imported, the work area is delimited and the type of tissue is selected by means of the upper and lower threshold values, which make it possible to distinguish different types of biological tissues (Figure 1).

This program has a set of tools capable of generating 3D models in the format ^*∗*^.STL (standard triangulation language) from a scan (tomography). Such a tool is the threshold used for carrying out the process of image segmentation. The threshold is a computational algorithm that segments structures automatically based on the definition of density ranges expressing grey pixels that relate to the tissue of interest. The main objective is to identify the pixels belonging to the structure and discriminate unwanted adjacent structures, resulting in the identification of each tissue across the image.

After segmentation, the program recognizes defined areas in each section from the volume of these images given in each section, one over the other and it generates a virtual model that is a reference for the solid model. In this case, to distinguish different molar tissues (the enamel, dentin, and pulp), object segmentation was performed as follows. Each segmentation contains only the pixels in the image with a value greater than or equal to the sill value. Therefore, it is necessary to have an upper and a lower threshold value. The selected objects contain all the pixels between these two values. A low threshold value corresponds to soft tissue and a high threshold to dense bone tissue or parts, which is possible by means of a histogram that shows the Hounsfield units (HU).

The HU can identify the type of tissue that is being observed (the bone, cartilage, calcification, enamel, dentin, and pulp); this distinction is made through the observation of the whole tomographic volume. For this, it is necessary to define the grey scale. The grey scale goes from black to white, with values between 0 and 255, considering 0 as black and 255 as white.

The values between 240 and 234 correspond to the cortical bone. The values between 190 and 230 correspond to the trabecular bone. The values between 83 and 112 are for the cartilaginous tissue. The value of 225 is for the enamel. The values between 198 and 237 are for the dentin. Finally, the value of 0 identifies the dental pulp [32]. Once these thresholds are identified, it proceeds to perform the modelling of each tissue by independent masks (each with its own colour identification). Several masks are required to obtain a final segmentation containing the required information (using working axes *X*, *Y*, and *Z*).

**Figure 2:**
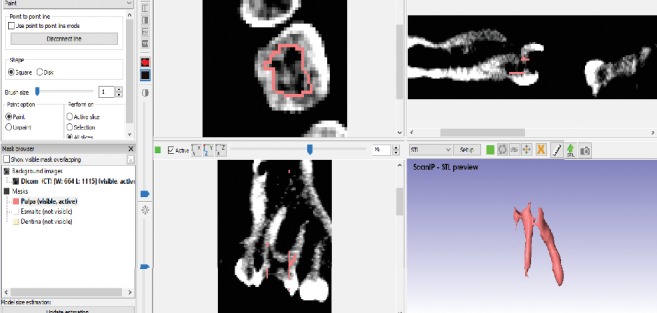
Delimitation of the pulp.

**Figure 3:**
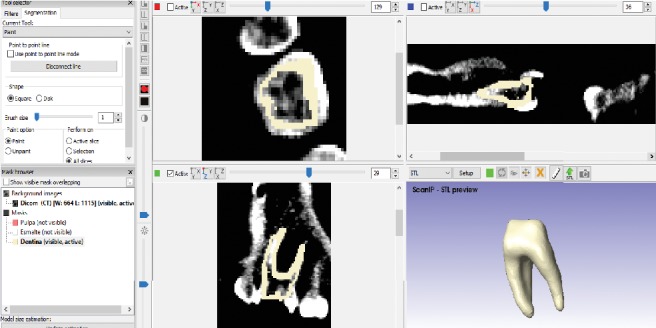
Delimitation of the dentin.

**Figure 4:**
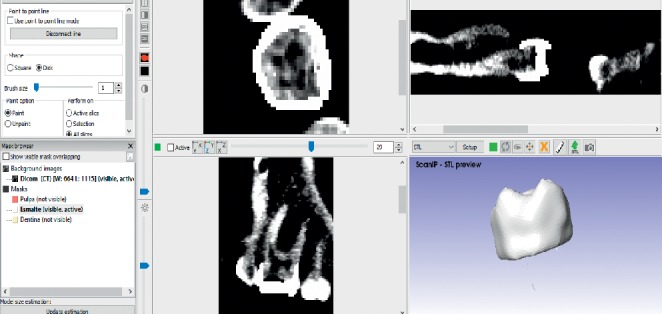
Delimitation of the enamel.

Once this is done, the working window is displayed where it is possible to see the three views of the tomographies (frontal, transversal, and sagittal) and a fourth that simulates the 3D view. When this view is obtained, it is possible to wiggle the cross-sectional areas in order to have a delimited space, for each tissue of the tooth. Once the space is delimited, the contours generated in the previous step are filled in to have a specific area for each tissue (Figures 2–4).

**Figure 5:**
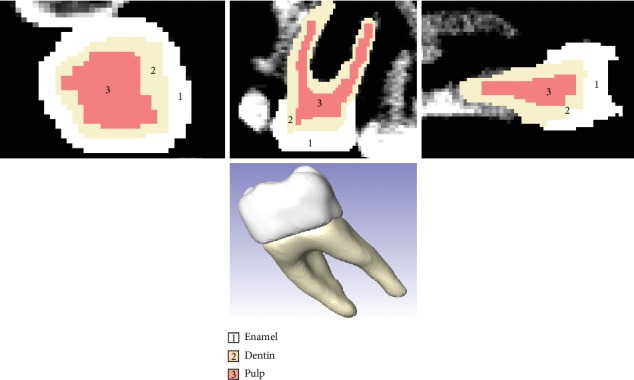
Finished model from molar ScanIP®.

**Figure 6:**
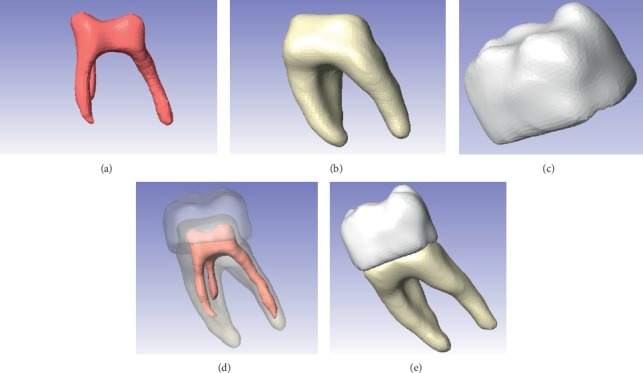
Biomodels obtained by the application of the ScanIP® program. (a) Dental pulp, (b) dental roots, (c) dental crown, (d) molars forming internal structure, and (e) molars forming external structure.

The tooth body was modelled with three tissues, each in its corresponding anatomical position (Figure 5). This procedure is repeated to all courts that comprise the area of interest. When filling or drawing the area, in a sequence of overlapping layers, it generates a series of points that, when placed on top of another, the three-dimensional model begins to be generated. In this way, different tissues are needed, producing new masks in the archive [33]. After the identification of each mask, ^*∗*^.STL formats for each dental organ tissue were generated. One model with three different elements, pulp, dentin, and enamel, was obtained (Figure 6).

After generating ^*∗*^.STL formats, it is possible to export the model in order to analyse it using CAD computer programs. The main objective of this is to convert ^*∗*^.STL model to a model with closed surfaces and make it a solid body. This must be done and is achieved by obtaining a hollow-shell type surface called point cloud. In addition, the phases of segmentation and 3D reconstruction, as well as the imported geometry to the CAD program, as there may be imperfections in the surface of the biomodel, an analysis and correction of triangulation or geometric mesh generated should be performed.

In this case, the program PowerShape® was applied. This is a computer program designed for the purpose of reverse engineering, in which from scanning of pieces, which generate point clouds that become geometries capable of working with any CAD computational tool, is performed.

**Figure 7:**
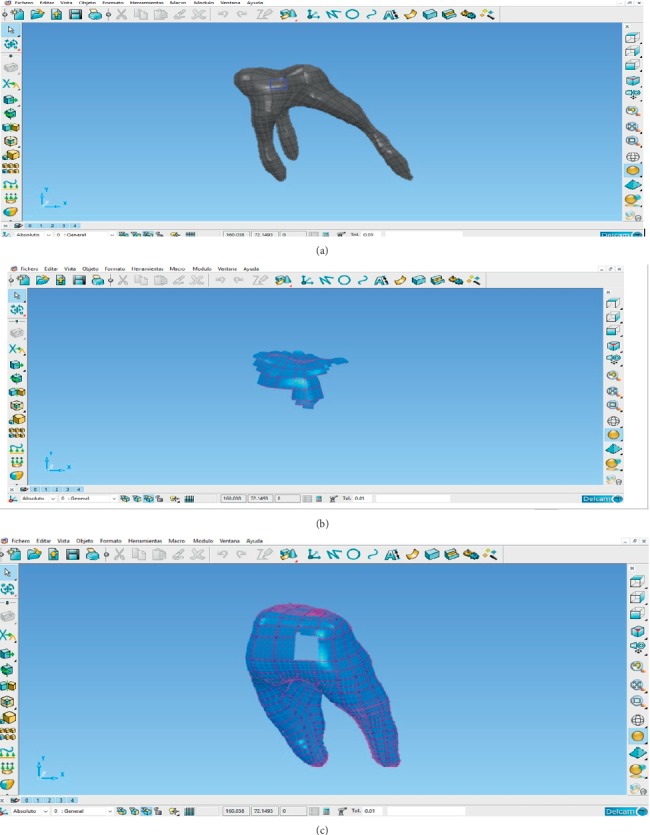
Meshing of the model in PowerShape®.

This program has tools that can scan the surface for image defects and fix them automatically or manually (Figure 7). With this program, the parametric surfaces were developed by manually discretizing to identify errors in the imported, such as sharp angles, fragmented lines, and overlapping points. If these errors are not corrected, discontinuities could arise between the nodes, which would not allow for final discretization and thus the analysis of the model. This step was performed separately for each molar tissue.

Once the discretized model is obtained, it is necessary to create a solid shape for data translation and create the IGES files (Initial Graphics Exchange Specification), which is the specification of initial exchange of graphics. This kind of files defines a neutral data format that allows the digital exchange of information between computer system designs (CADs). This makes it possible to export the model created in the ANSYS® program to perform the corresponding analysis.

**Figure 8:**
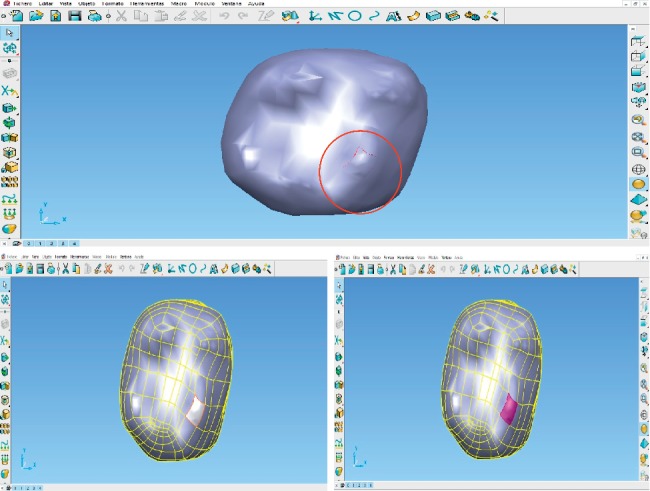
Correction of surfaces and solidification of the model.

In this case, the CopyCad® program was used to generate the solid shape. This program allows the conversion of discretized (mesh) into a solid. The application of this conversion tool geometry of the structure of the solid mesh allows the surface to become an easily editable solid entity, preparing for submission to the implementation of various CAD tools (Figure 8).

**Figure 9:**
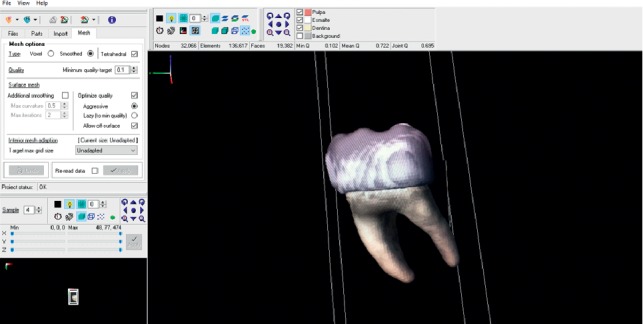
Assignment of mechanical properties of tissues.

When it is found that there are no errors in the geometry created, the model is exported to a program with CAD tools. The program used was ScanFE® in which the mechanical properties of the three materials which constitute the model were assigned, and it was possible to generate a mesh to perform analysis (Figure 9). Finally, the model obtained and transformed in an IGES or parasolid file is exported to the ANSYS® program. This was done in order to test the viability of the model for analysis on it.

## 3. Results and Discussion

**Figure 10:**
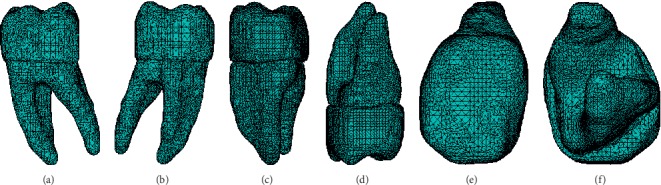
Biomodel imported into the ANSYS® program for its numerical simulation. (a) Vestibular face, (b) lingual face, (c) mesial face, (d) distal face, (e) occlusal face, and (f) cervical face.

The model was interpreted by the ANSYS® program as a structure consisting of three different materials (the enamel, dentin, and pulp), with a total of 118458 elements and 26217 nodes. The model was made by means of high-order elements (tetrahedral with a total of 10 nodes per element) (Figure 10).

The analysis is linear, elastic, and isotropic. Boundary conditions were settled at the roots of the molar. A restriction was made in all degrees of freedom. A 15 N load (pressure) at the occlusal surface of the molar, on the contact points, was applied. The mechanical properties of the tissues which were considered are density, Young's modulus, and dimensional Poisson's ratio. For the enamel, they are 0.25 g/cm^3^, 70 GPa, and 0.30. For the dentin, the values are 0.31 g/cm^3^, 18.3 GPa, and 0.30. Finally, for the pulp, they are 0.1 g/cm^3^, 2 GPa, and 0.45 [34].

In order to simulate the elastoplastic behaviour of each of the tissues, a general model was used, which is based on a kinematic hardening rule with isotropic hardening components comprising the following equations [35]:

(1) $$\begin{array}{c} \text{d}a=C\frac{1}{\sigma_{0}}\left(\sigma -\alpha \right)\text{d}{\bar{\varepsilon }}^{pl}-\gamma \alpha \text{d}{\bar{\varepsilon }}^{pl},\\ \sigma_{0}={\left.\sigma \right|}_{0}+Q_{\infty }\left(1-e^{-{\bar{\varepsilon }}^{pl}}b\right), \end{array}$$

where ${\bar{\varepsilon }}^{pl}$  = equivalent plastic unit deformation, *α* = effort of the lower surfaces, *C* = kinematic initial module, *γ* = range in which the kinematic module decreases with respect to the plastic deformation, *σ*_0_ = current transfer effort, *σ*|_0_  = original transfer effort, *Q*_*∞*_ = maximum change in yield surface size, and *b* = the range in which the yield surface changes in relation to the development of the plastic unit deformation.

**Figure 11:**
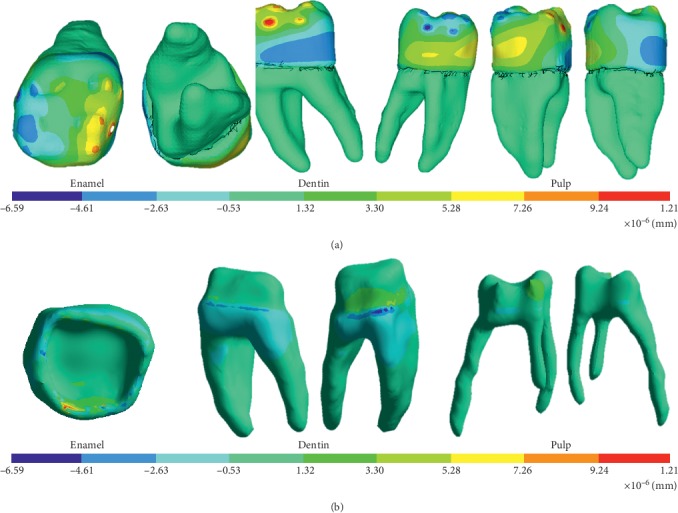
Directional displacement on the *X*-axis. (a) External structure of the biomodel. (b) Internal structure of the biomodel.

**Figure 12:**
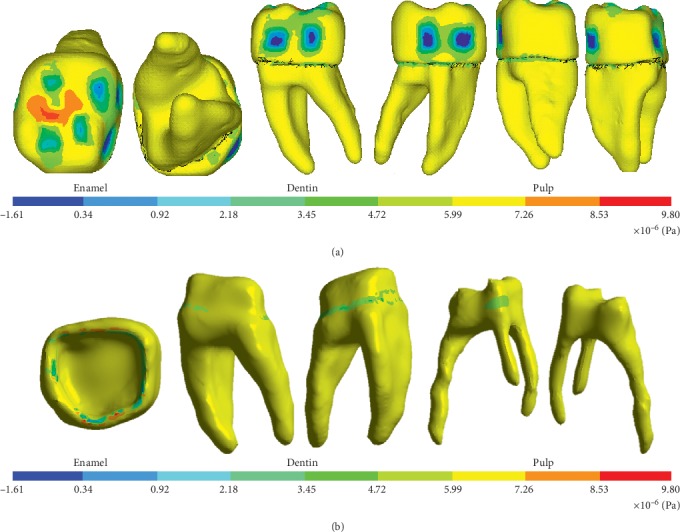
Normal stresses on the *Y*-axis. (a) External structure of the biomodel. (b) Internal structure of the biomodel.

**Figure 13:**
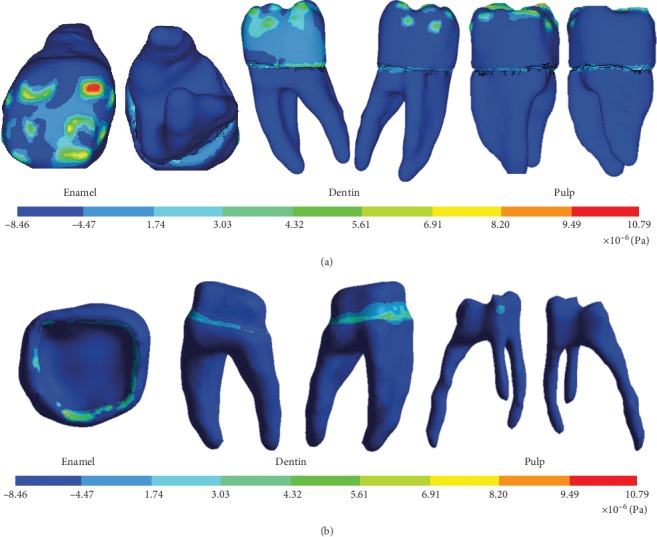
Shear stresses on the *X*-axis. (a) External structure of the biomodel. (b) Internal structure of the biomodel.

The model was viable, since results such as the deformed shape, general displacement, and displacement in the *X*, *Y*, and *Z* axes were obtained. Nominal stresses in all the three axes are first principal stresses, von Mises stresses, and shear stresses (Figures 11–13).

The first biomodels of dental organs were obtained by scanning dental plaster models to obtain the geometry of the dental organ. Subsequently, those that use the conventional microtomography of some extracted dental organs were developed. Unfortunately, with these methodologies, it is not possible to faithfully reproduce the morphology and, therefore, specific characteristics of it were not reproduced faithfully (it is not possible to have an adequate control of the limits of the amelo-dentinal union, among others).

The contact zone between the enamel and the dentin is not a smooth and regular area. It is described as an irregular scalloped border, where you can see dentin protrusions projecting towards the enamel. The dentin being formed by mineralized collagen and hydroxyapatite cells is not formed independently of enamel but is integrated to the own. This causes an irregular boundary between both tissues to be established.

The results show that the methodology proposed in this work allows to solve the previously mentioned situations. The obtained biomodel faithfully reproduces the external and internal morphology of the dental organ. The anatomy obtained is highly detailed both in its external and internal geometry since it is structured with the three tissues that make them up. It also has the mechanical properties of each of them, which allows having a solid composed of three different materials, the situation that was not considered in previously applied methodologies.

In addition to this, this methodology allows three-dimensional analysis. The previous methodologies only allowed two-dimensional analysis, which leads to the results being far from reality. They allow the possibility of predicting risk zones for future ones, both in healthy teeth and in teeth with a history of caries and being restored, and in the same way to indicate the weakened areas of the remaining dental tissues, where the onset of faults can occur. In the results obtained, it is clearly observed that these areas should be considered since they are also of great interest in the clinical part.

The methodology described results in the generation of three-dimensional models of high biofidelity, with a clear differentiation of the tissues with their specific properties, which makes it somewhat complicated to be able to have comparison parameters with methodologies, which, as already mentioned, do not have these advantages.

## 4. Conclusions

Development of Biomodels of dental organs is a difficult task because of the complex morphology, the characteristics of heterogeneous materials, anisotropic condition, nonlinearity, and viscoelastic parameters. There are several studies describing procedures for the development of such models. These studies emphasize that although this method is a powerful tool, one should be careful in the methodology for creating them [36, 37]. This led to the proposal of different methodologies. Conventional tomography basis of macroplaster models has been used for obtaining dental body geometry [35, 38]. Another methodology pre-existing to the proposal in this work used the conventional tomography of dental extractions of a patient so that its density changed and the geometry lost its biofidelity [39]. These are based on the methods developed for producing solid models of the bone. These models used the *Hounsfield* unit (HU) scan density. However, it was not possible to have proper control of the limits for the dentine-enamel junction [40–42].

The methodology proposed in this work can solve the aforementioned situations. The biomodel obtained faithfully reproduced the external and internal morphology of the dental organ. Anatomy obtained is very detailed both in its external and internal geometry, as it is structured with the three tissues that form it. It also has the mechanical properties of each of them, which allows a solid consisting of three different materials. All these qualities of the biomodel are due to the fact that, in the methodology, the three main factors mentioned at the beginning of the manuscript are considered: the quality of the DICOM images (when using a novel imaging technique such as the TVC), have the necessary training for the proper management of computer programs, and knowledge of the morphology of the specific area to be modeled. For this, the multidisciplinary between Dentistry, Engineering, and Biomechanics was necessary.

Nevertheless, there is a relevant aspect that should be considered, the amount of time required for designing and obtaining the necessary volumetric image. Biomodel fidelity is directly related to the time factor. This coupled with the knowledge of computer programs is necessary; although they are not a complicated issue, they require the operator to receive training and education of management. Furthermore, it is necessary to use various programs to ensure the quality of the obtained model, which increases the time factor. In addition, the import and export models from one program to another could itself cause discrepancies between program generation communication failures.

**Figure 14:**
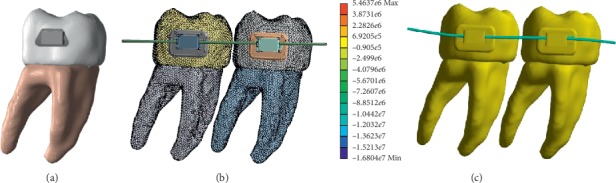
Biomodel generated with the proposed methodology, for lingual orthodontic technique: (a) molar and bracket, (b) assembly of molars with bracket-wire system, and (c) numerical analysis of the stress generated in the wire.

Nonetheless, the methodology itself does not require programs and sophisticated computer equipment. The biomodel obtained from such a methodological approach is the basis for the development of various kinds of analyses that, given the nature of the biomodel, allows the simulation of various diseases, treatments, fixtures, etc (Figure 14).

Compared to other biomodels presented in other studies, the proposed model has a much better defined morphology and contemplates different properties of all the tissues that make up the dental organ. The study carried out by Da Silva [43], where the faithful morphology of an incisor tooth was obtained, takes into account all the tissues of a molar. The mechanical properties of each material were taken from the literature and are the same as those taken for the present work, as well as border conditions and the same range of applied loads.

The results of the analysis were compared with those obtained in the study of Uddanwadiker [42] (Table 1). The difference between them is due to the increased morphological model accuracy obtained in this work, and the model has specific mechanical properties of each tissue, allowing it to be closer to the reality of the masticatory process.

Due to the morphological, morphometric precision, and the information of the mechanical properties that the model has, it can be used for various analyzes and studies. It is only necessary to change the boundary conditions, the application of forces, restrictions, mechanical properties, eliminate any area, and so on. In this way, it is only necessary to make a few changes, so several simulations, numerous analyses, and infinite possibilities can be driven from this kind of biomodel. Various disorders, diseases, and treatments can be well analysed with this biomodel personalized for each patient, enabling better diagnoses and plans best suited to the circumstances of each patient treatment, reducing the costs and risks.

**Figure 15:**
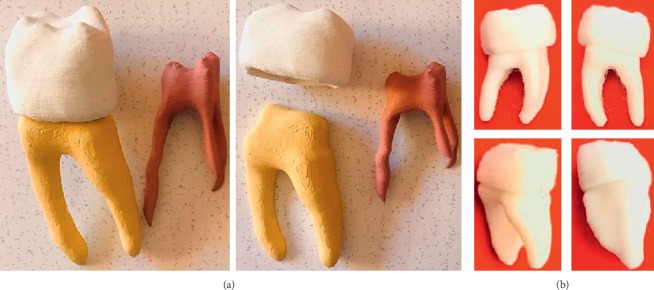
3D printing of molar biomodel through the proposed methodology: (a) scale 5 : 1 and (b) real size.

One more advantage of the methodology described in this work is that it has the possibility of being applied not only in any dental organ but it can also be used for any biological tissue or organ, whether human, animal, or vegetable. In this work, a tiny organ is used to describe the methodology, so it has the possibility of generating biomodels of larger structures, such as long bones or bigger and bulky organs. In addition, the biomodels generated through this methodology can be implemented for 3D printing entirety or for the printing of restorations, with total morphological and morphometric attachments (Figure 15).

## Figures and Tables

**Table 1:** Comparative table of the results obtained applied to the biomodels of the Uddanwadiker study and the one proposed in this work.

	Displacement (mm)	Principal stresses (MPa)	von Mises stresses (MPa)
Uddanwadiker et al. [42]	0.00026	−19 to 10	0.7 to 25
Proposed methodology	0.00035	−15 to 16.07	0 to 27.05

## Data Availability

The data used to support the findings of this study are included within the article.
